# PI3K/AKT/PTEN Signaling as a Molecular Target in Leukemia Angiogenesis

**DOI:** 10.1155/2012/843085

**Published:** 2012-02-28

**Authors:** Naoko Okumura, Hitomi Yoshida, Yasuko Kitagishi, Mutsumi Murakami, Yuri Nishimura, Satoru Matsuda

**Affiliations:** Department of Environmental Health Science, Nara Women's University, Kita-Uoya Nishimachi, Nara 630-8506, Japan

## Abstract

PI3K/AKT/PTEN pathway is important in the regulation of angiogenesis mediated by vascular endothelial growth factor in many tumors including leukemia. The signaling pathway is activated in leukemia patients as well as leukemia cell lines together with a decrease in the expression of PTEN gene. The mechanism by which the signaling pathway regulates angiogenesis remains to be further elucidated. However, it has become an attractive target for drug therapy against leukemia, because angiogenesis is a key process in malignant cell growth. In this paper, we will focus on the roles and mechanisms of PI3K/AKT/PTEN pathway in regulating angiogenesis.

## 1. Introduction

**Figure 1:**
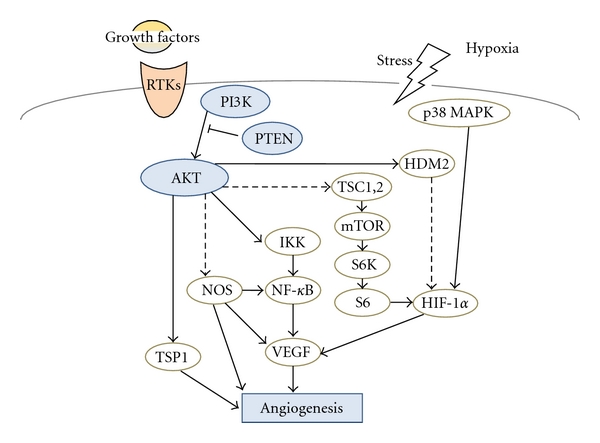
Schematic representation of PI3K/AKT/PTEN signaling. Examples of molecules known to act on angiogenesis via PI3K/AKT regulatory pathways are shown.

Angiogenesis is the process by which new blood capillaries are generated from the preexisting blood vessels [1, 2]. Tumor angiogenesis is essential for tumor growth, invasion, and metastasis [3, 4]. This process can be triggered by a series of signal pathways including extracellular signals such as growth factors (Figure 1). It is a complex process that is also regulated by pro- and antiangiogenic factors. In other words, the angiogenesis and vasculature are regulated through the change of balance between the collective actions of proangiogenic factors such as vascular endothelial growth factor (VEGF) and angiogenic inhibitors such as thrombospondin-1(TSP-1). These factors can be derived from different sources such as stromal cells, extracellular matrix, and cancer cells. Their relative contribution is likely to be different according to the difference in tumor types. The interaction btween cancer cells and vascular endothelial cells in the tumor microenvironment affects the angiogenesis [5, 6]. Leukemia is an aggressive malignancy characterized by the accumulation of immature leukemia blasts in the bone marrow. Bone marrow angiogenesis is therefore important for both leukemogenesis, and the leukemic bone marrow shows increased microvascular density [7].

VEGF and VEGF receptor (VEGFR) are major angiogenesis inducer associated with tumor angiogenesis in numerous solid or hematological malignancies. VEGF binds to VEGF receptor, which leads to the activation of phosphatidylinositol 3-kinase (PI3K)/Akt signaling pathway. In addition to the PI3K/Akt signaling, phosphatase and tensin homolog deleted on chromosome 10 (PTEN) play an important role as a molecular inhibitor of PI3K/Akt signaling in multiple cellular functions such as cell proliferation, cell-cycle progression, and survival [8]. PI3K/Akt signaling regulates angiogenesis through affecting the expression of VEGF (Figure 1). It may contribute to tumor angiogenesis not only via the autocrine pathway to tumor cells but also via a paracrine pathway to the surrounding microvessels. The amplification and mutations of PI3K/Akt and the loss of the tumor suppressor PTEN are common in various kinds of human tumors including leukemia. In addition, the activation of PI3K/Akt signaling is commonly observed in numerous leukemia patients and leukemia cell lines together with a decrease in the expression of PTEN [9]. As siRNA against PI3K and Akt greatly decreases tumor growth and angiogenesis [10], it is considered that PI3K/Akt pathways indeed involved in the tumor angiogenesis. In this paper, we will focus on the roles and mechanisms of PI3K, AKT, and PTEN in regulating angiogenesis and roles of the downstream targets for transmitting the signals. 

## 2. Function of PI3K/AKT in Angiogenesis

**Figure 2:**
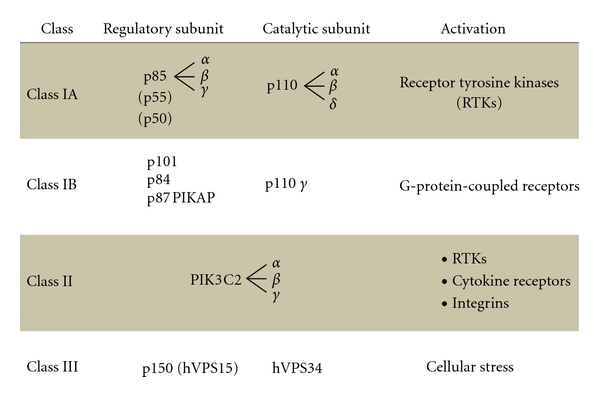
Representation of subclass molecules of PI3K. PI3K is composed of three subclasses based on the substrate, structure, distribution, and mechanism of activation.

The active form of PI3K is an oncogene, and amplifications and mutations of PI3K are commonly found in many kinds of human cancers [11]. Genetic alterations of PI3K lead to dysfunction of vasculature and angiogenesis. In addition, forced expression of PI3K alone is sufficient to increase angiogenesis via increased VEGF expression [12]. The PI3K in mammalian cells forms a family that can be divided into three classes based on their structure, distribution, and mechanism of activation (Figure 2). Class I PI3Ks are divided into class IA and class IB based on different associated adaptors. Class IA PI3Ks are activated by receptor tyrosine kinases, while class IB PI3Ks are activated by G-protein-coupled receptors. These PI3Ks are heterodimers consisting of a regulatory subunit such as p85 and a catalytic subunit such as p110. The p110 is required to control endothelial cell migration and angiogenesis, and p110-knockout endothelial cells lead to embryonic lethality with severe defects in angiogenic sprouting and vascular remodeling [13]. The phospholipid second messengers generated by PI3K provide a common mechanism for multiple steps during angiogenesis. PI3K inhibitor LY294002 decreased tumor-induced angiogenic response [14].

**Figure 3:**
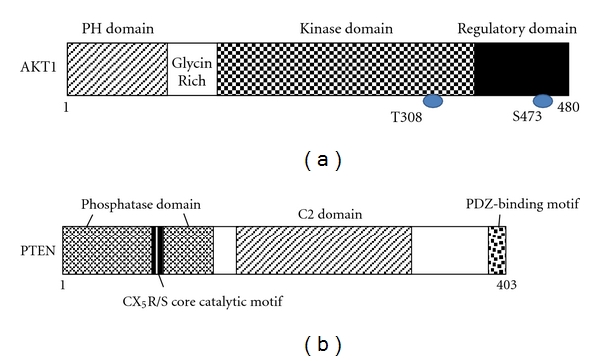
Schematic structures of AKT1 (a) and PTEN (b) protein. The predicted consensual domain structures for each protein are depicted. PH domain: pleckstrin homology domain; C2 domain: a protein structural domain involved in targeting proteins to cell membranes; PDZ: a common structural domain in signaling proteins (PSD95, Dlg, ZO-1, etc.).

Serine-threonine protein kinase AKT (also known as protein kinase B) is a major downstream target of PI3K for regulating tumor growth and angiogenesis. AKT is initially found to be the cellular homolog of AKT8 retroviral oncogene [15]. Human AKT has three isoforms: AKT1, AKT2, and AKT3. PIP3, a product of PI3K, binds to AKT and leads to the membrane recruitment of AKT and also binds to phosphoinositide-dependent kinase 1 (PDK1) via their pleckstrin homology (PH) domains, and then PDK1 phosphorylates AKT in the kinase domain (Thr 308 in AKT1). For the full activation of AKT, the phosphorylation within the carboxyl-terminal regulatory domain (Ser 473 in AKT1) of AKT by PDK2 is required [16]. Schematic structure of the predicted AKT1 protein is shown in Figure 3(a). Once activated, AKT moves to the cytoplasm and nucleus, where it phosphorylates, activates, or inhibits many downstream targets to regulate various cellular functions including angiogenesis. The forced expression of active forms of PI3K/Akt increases the number of sprouting vessels to induce angiogenesis. Bone-marrow-derived endothelial cells and some hematopoietic progenitors participate in the angiogenesis. AKT can activate NF-**κ**B pathway [17], performing a complicated network in regulating angiogenesis (Figure 1). Transgenic expression of Myr-AKT in endothelial cells is sufficient to form the structural and functional features of blood vessels [18]. The sustained endothelial AKT activation causes enlarged blood vessels and its effect can be reversed by the AKT inhibition. AKT inhibits the GTPase-activating protein (GAP) activity of the tuberous sclerosis complex 1 (TSC1) and TSC2 complex by phosphorylating TSC2 tuberin protein, leading to the accumulation and activation of the mTOR and raptor complex [19]. The mTOR mediates the phosphorylation of the ribosomal protein S6 kinases and eukaryotic translation initiation factor 4E-binding protein 1 leading to the release of the translation initiation factor eIF4E [20].

## 3. Function of PTEN in Angiogenesis

PTEN is a dual-specificity phosphatase which has protein phosphatase activity and lipid phosphatase activity that antagonizes PI3K activity [21]. PTEN gene, which encodes 403-residue amino acids, is located on chromosome 10q23.3. Schematic structure of the predicted PTEN protein is shown in Figure 3(b). PTEN negatively regulates the activity of PI3K/Akt signaling through converting phosphatidylinositol 3,4,5-triphosphate (PIP3) into phosphatidylinositol 4,5-bisphosphate (PIP2). Because PTEN protein plays an important role in regulating proliferation and invasion of many cancer cells, PTEN is considered as a tumor suppressor. PTEN also modulates angiogenesis via down-regulating PI3K/Akt pathway in many tumors including leukemia [22–24]. Although the effects of PTEN on invasion of hematopoietic cells and its clinical significance remain to be further elucidated, PTEN would be a candidate target to be addressed for inhibiting angiogenesis along with the treatment of leukemia [25]. Recent study has demonstrated that in addition to suppressing AKT activation, PTEN also controls the activity of Jun N-terminal kinase (JNK) [26]. PTEN-knockout endothelial cells cause embryonic lethality due to endothelial cell hyperproliferation and impaired vascular remodeling, whereas PTEN+/− endothelial cells enhance neovascularization and tumor angiogenesis to increase tumor growth [27]. As PTEN is frequently mutated or lost in a number of human cancers, PTEN can be upregulated by early-growth-regulated transcription factor 1 (EGR1) through direct binding to the PTEN promoter [28]. In addition, peroxisome-proliferator-activated receptor **γ**(PPAR**γ**), p53, and activating transcription factor 2 (ATF2) can also transcriptionally upregulate PTEN [29, 30], while transforming growth factor (TGF)-**β**, nuclear factor kappaB (NF-**κ**B), and Jun negatively regulate PTEN expression [31, 32]. Interestingly, rosemary extract represses PTEN expression in K562 leukemic culture cells [33]. Some microRNAs such as miR-21, miR-19a, and miR-214 inhibit PTEN through targeting the 3′-untranslated region of PTEN, leading to inhibition of PTEN translation [34]. PTEN activity can also be regulated by the posttranslational regulation including phosphorylation, acetylation, and oxidation.

## 4. Downstream Molecules Mediated by PI3K/AKT/PTEN in Regulating Angiogenesis

PI3K/Akt signaling pathway induces tumor growth through the expression of angiogenic factors and the inhibition of antiangiogenic molecules. PI3K/Akt and their effectors, hypoxia-inducible factor-1**α** (HIF-1**α**) and VEGF, play key roles in regulating the angiogenesis [35, 36]. PI3K/Akt may also regulate angiogenesis by several downstream targets such as mTOR/p70S6K1, FOXO, NOS, and GSK-3**β**. These targets commonly upregulate HIF-1**α**expression which induces VEGF transcriptional activation. Inhibition of GSK-3**β** can upregulate HIF-1**α**expression and increase **β**-catenin activity [37]. Hypoxia induces HIF-1**α**production through the increase of its stability and induces VEGF expression in a HIF-1-dependent manner. PI3K can also induce VEGF expression through HIF-1**α**and NF-**κ**B activation. PI3K/Akt can suppress TSP1, the endogenous antiangiogenic molecule, in both cancer cells and endothelial cells [38]. The TSP1 is a family member of TSP proteins with potent antiangiogenic activity. TSP1 inhibits angiogenesis endothelial cell proliferation and migration. In contrast, TSP1 is an important autocrine factor for vascular smooth muscle cell proliferation and migration. AKT1-knockout mice showed impaired vascular maturation with decreased expression of TSP-1 and TSP-2, while reexpression of TSP-1 and TSP-2 in mice transplanted with wild-type bone marrow is associated with the angiogenesis [39]. The endothelial NOS (eNOS) is critical for VEGF-triggered postnatal angiogenesis [40, 41]. Several protein kinases, such as Akt, AMP-activated protein kinase (AMPK), and protein kinase A (PKA), are known to activate eNOS [42]. Among them, Akt has emerged as a central regulator for eNOS activation by VEGF. Inhibition of Akt activity impairs the phosphorylation of the human homologue of murine double minute-2 (HDM2), resulting in the destabilization of HDM2 [43]. It is known that Akt-dependent phosphorylation of HDM2 causes nuclear translocation of HDM2 followed by HDM2-mediated inactivation of p53. Overexpression of p70S6K1 in microvascular endothelial cells enhanced tumor growth and angiogenesis, while HIF-1**α**siRNA significantly inhibited tumor growth and angiogenesis, suggesting that endothelial p70S6K1 controls tumor angiogenesis through HIF-1**α** [44].

## 5. Inhibitors Involved in PI3K/AKT Signaling

Pan-PI3K inhibitors were initially discovered; however, isoform-specific PI3K inhibitors have less toxicity to the cells than pan-PI3K inhibitors, which could be used to specifically target PI3K activation in certain cancer cells. Pan-PI3K inhibitors, wortmannin and LY294002, are commonly used to inhibit cancer cell proliferation and tumor growth [45]. Wortmannin is a fungal product, which exerts its effect by the covalent interaction to the conserved Lys802 of the p110**α**  catalytic subunit. Both wortmannin and LY294002 also cross-react with PI3K-related kinases such as mTOR and DNA-dependent protein kinases. These inhibitors have poor solubility and high toxicity because they target a broad range of PI3K-related enzymes. A novel pegylated 17-hydroxywortmannin (PWT-458) is watersoluble and has shown improvements in drug stability [46]. A p110**δ**-specific inhibitor (IC486068) enhances radiation-induced tumor vascular destruction [47].

The first developed group of AKT inhibitors was lipid-based inhibitors that include perifosine, phosphatidylinositol ether lipid analogs (PIAs), and D-3-deoxy-phosphatidylmyoinositol-1-[(R)-2-methoxy-3-octadecyloxyropyl hydrogen phosphate] (PX-316), which showed antitumor effects. Perifosine inhibits the translocation of AKT to the cell membrane [48]. Inositol (1,3,4,5,6) pentakisphosphate [Ins (1,3,4,5,6) P5], one of the PI3K/AKT inhibitors, also inhibits tumor growth and angiogenesis [49]. Several other AKT antagonists such as 9-methoxy-2-methylellipticinium acetate (API-59-OMe), indazole-pyridine A-443654, and isoform-specific canthine alkaloid analogs have been identified and shown to inhibit cancer cell growth and induce apoptosis. Other kinds of AKT inhibitors include peptide-based inhibitors of AKT, pseudopeptide substrates of AKT, a single-chain antibody against AKT, an inhibitory form of AKT mutant, and siRNA, against AKT.

The mTOR inhibitors such as rapamycin and its analogs inhibit mTOR activation by binding to FK506-binding protein-12 (FKBP12) [50]. There is a feedback loop because p70S6K1 negatively regulates insulin receptor substrate and PDGF receptor. Rapamycin or its analogs can activate upstream molecules including AKT due to the loss of feedback inhibition. It is important to exploit the potential benefits of the targeted therapies and optimal treatment with these inhibitors.

## 6. Perspective

The bone marrow of the leukemia patients has increased blood vessel content compared to normal counterparts, suggesting that leukemia progression might be accompanied with an increase of vascularization and suggesting the possibility for a role of antiangiogenic therapy in the treatment of leukemia. PI3K/Akt/PTEN signaling regulates angiogenesis through the interaction of cancer cells and tumor microenvironments including endothelial cells. Angiogenesis inducers such as VEGF can activate PI3K/Akt signaling for inducing angiogenesis. Given the important role of the signaling pathway in regulating tumor growth and angiogenesis, development of therapeutic drugs using the PI3K/Akt signaling inhibitors becomes important for cancer treatment. In addition, improving the function of PTEN offers another approach for targeting angiogenesis and apoptosis induction, which could be important for the development of leukemia therapeutics [51]. PI3K/Akt in turn regulates tumor growth and angiogenesis through downstream targets, mTOR, p70S6K1, HIF-1, and VEGF. Their upstream and downstream molecules are commonly altered in human cancers and play an important role in angiogenesis. Accordingly, PI3K/Akt pathway inhibitors are likely more effective in patients with active PI3K/Akt signaling in case such as PTEN mutations. The therapeutic methods targeting PI3K/Akt/PTEN pathway would represent the promising leukemia therapy in the future.

## References

[1] Piyaviriyakul S, Shimizu K, Asakawa T, Kan T, Siripong P, Oku N (2011). Anti-angiogenic activity and intracellular distribution of epigallocatechin-3-gallate analogs. *Biological and Pharmaceutical Bulletin*.

[2] Arnaoutova I, Kleinman HK (2010). In vitro angiogenesis: endothelial cell tube formation on gelled basement membrane extract. *Nature Protocols*.

[3] Zhang Y, Tang H, Cai J (2011). Ovarian cancer-associated fibroblasts contribute to epithelial ovarian carcinoma metastasis by promoting angiogenesis, lymphangiogenesis and tumor cell invasion. *Cancer Letters*.

[4] Rouhi P, Lee SLC, Cao Z, Hedlund EM, Jensen LD, Cao Y (2010). Pathological angiogenesis facilitates tumor cell dissemination and metastasis. *Cell Cycle*.

[5] Bruni-Cardoso A, Johnson LC, Vessella RL, Peterson TE, Lynch CC (2010). Osteoclast-derived matrix metalloproteinase-9 directly affects angiogenesis in the prostate tumor-bone microenvironment. *Molecular Cancer Research*.

[6] Schmid SA, Gaumann A, Wondrak M (2007). Lactate adversely affects the in vitro formation of endothelial cell tubular structures through the action of TGF-*β*1. *Experimental Cell Research*.

[7] Alexandrakis MG, Passam FH, Pappa CA (2005). Serum evaluation of angiogenic cytokines basic fibroblast growth factor, hepatocyte growth factor and TNF-alpha in patients with myelodysplastic syndromes: correlation with bone marrow microvascular density. *International Journal of Immunopathology and Pharmacology*.

[8] Jiang BH, Liu LZ (2009). PI3K/PTEN signaling in angiogenesis and tumorigenesis. *Advances in Cancer Research*.

[9] Shafee N, Kaluz S, Ru N, Stanbridge EJ (2009). PI3K/Akt activity has variable cell-specific effects on expression of HIF target genes, CA9 and VEGF, in human cancer cell lines. *Cancer Letters*.

[10] Xia C, Meng Q, Cao Z, Shi X, Jiang BH (2006). Regulation of aneiogenesis and tumor growth by p110 alpha and AKT1 via VEGF expression. *Journal of Cellular Physiology*.

[11] Courtney KD, Corcoran RB, Engelman JA (2010). The PI3K pathway as drug target in human cancer. *Journal of Clinical Oncology*.

[12] Skinner HD, Zheng JZ, Fang J, Agani F, Jiang BH (2004). Vascular endothelial growth factor transcriptional activation is mediated by hypoxia-inducible factor 1*α*, HDM2, and p70S6K1 in response to phosphatidylinositol 3-kinase/AKT signaling. *Journal of Biological Chemistry*.

[13] Graupera M, Guillermet-Guibert J, Foukas LC (2008). Angiogenesis selectively requires the p110*α* isoform of PI3K to control endothelial cell migration. *Nature*.

[14] Su JD, Mayo LD, Donner DB, Durden DL (2003). PTEN and phosphatidylinositol 3′-kinase inhibitors up-regulate p53 and block tumor-induced angiogenesis: evidence for an effect on the tumor and endothelial compartment. *Cancer Research*.

[15] Staal SP (1987). Molecular cloning of the akt oncogene and its human homologues AKT1 and AKT2: amplification of AKT1 in a primary human gastric adenocarcinoma. *Proceedings of the National Academy of Sciences of the United States of America*.

[16] Kroner C, Eybrechts K, Akkerman JWN (2000). Dual regulation of platelet protein kinase B. *Journal of Biological Chemistry*.

[17] Tanaka H, Fujita N, Tsuruo T (2005). 3-Phosphoinositide-dependent protein kinase-1-mediated I*κ*B kinase *β*(IKKB) phosphorylation activates NF-*κ*B signaling. *Journal of Biological Chemistry*.

[18] Jiang BH, Zheng JZ, Aoki M, Vogt PK (2000). Phosphatidylinositol 3-kinase signaling mediates angiogenesis and expression of vascular endothelial growth factor in endothelial cells. *Proceedings of the National Academy of Sciences of the United States of America*.

[19] Tee AR, Manning BD, Roux PP, Cantley LC, Blenis J (2003). Tuberous Sclerosis Complex gene products, Tuberin and Hamartin, control mTOR signaling by acting as a GTPase-activating protein complex toward Rheb. *Current Biology*.

[20] Hagner PR, Schneider A, Gartenhaus RB (2010). Targeting the translational machinery as a novel treatment strategy for hematologic malignancies. *Blood*.

[21] Planchon SM, Waite KA, Eng C (2008). The nuclear affairs of PTEN. *Journal of Cell Science*.

[22] Bonnet M, Loosveld M, Montpellier B (2011). Posttranscriptional deregulation of MYC via PTEN constitutes a major alternative pathway of MYC activation in T-cell acute lymphoblastic leukemia. *Blood*.

[23] Martins LR, Lúcio P, Silva MC (2010). Targeting CK2 overexpression and hyperactivation as a novel therapeutic tool in chronic lymphocytic leukemia. *Blood*.

[24] Gutierrez A, Sanda T, Grebliunaite R (2009). High frequency of PTEN, PI3K, and AKT abnormalities in T-cell acute lymphoblastic leukemia. *Blood*.

[25] Steelman LS, Franklin RA, Abrams SL (2011). Roles of the Ras/Raf/MEK/ERK pathway in leukemia therapy. *Leukemia*.

[26] Vivanco I, Palaskas N, Tran C (2007). Identification of the JNK signaling pathway as a functional target of the tumor suppressor PTEN. *Cancer Cell*.

[27] Suzuki A, Hamada K, Sasaki T, Mak TW, Nakano T (2007). Role of PTEN/PI3K pathway in endothelial cells. *Biochemical Society Transactions*.

[28] Baron V, Adamson ED, Calogero A, Ragona G, Mercola D (2006). The transcription factor Egr1 is a direct regulator of multiple tumor suppressors including TGF*β*1, PTEN, p53, and fibronectin. *Cancer Gene Therapy*.

[29] Harikumar KB, Aggarwal BB (2008). Resveratrol: a multitargeted agent for age-associated chronic diseases. *Cell Cycle*.

[30] Xing LW, Zhang L, Youker K (2006). Free fatty acids inhibit insulin signaling-stimulated endothelial nitric oxide synthase activation through upregulating PTEN or inhibiting Akt kinase. *Diabetes*.

[31] Chow JYC, Ban M, Wu HL (2010). TGF-*β* downregulates PTEN via activation of NF-*κ*B in pancreatic cancer cells. *American Journal of Physiology*.

[32] Moore-Smith L, Pasche B (2011). TGFBR1 signaling and breast cancer. *Journal of Mammary Gland Biology and Neoplasia*.

[33] Yoshida H, Okumura N, Kitagishi Y, Nishimura Y, Matsuda S (2011). Ethanol extract of Rosemary repressed PTEN expression in K562 culture cells. *International Journal of Applied Biology and Pharmaceutical Technology *.

[34] Pezzolesi MG, Platzer P, Waite KA, Eng C (2008). Differential expression of PTEN-targeting microRNAs miR-19a and miR-21 in cowden syndrome. *American Journal of Human Genetics*.

[35] Gómez-Raposo C, Mendiola M, Barriuso J, Casado E, Hardisson D, Redondo A (2009). Angiogenesis and ovarian cancer. *Clinical &amp; Translational Oncology*.

[36] Ellis L, Hammers H, Pili R (2009). Targeting tumor angiogenesis with histone deacetylase inhibitors. *Cancer Letters*.

[37] Li Q, Michaud M, Canosa S, Kuo A, Madri JA (2011). GSK-3*β*: a signaling pathway node modulating neural stem cell and endothelial cell interactions. *Angiogenesis*.

[38] Pallero MA, Elzie CA, Chen J, Mosher DF, Murphy-Ullrich JE (2008). Thrombospondin 1 binding to calreticulin-LRP1 signals resistance to anoikis. *FASEB Journal*.

[39] Chen J, Somanath PR, Razorenova O (2005). Akt1 regulates pathological angiogenesis, vascular maturation and permeability in vivo. *Nature Medicine*.

[40] Kafousi M, Vrekoussis T, Tsentelierou E (2012). Immunohistochemical study of the angiogenetic network of VEGF, HIF1*α*, VEGFR-2 and endothelial nitric oxide synthase (eNOS) in human breast cancer. *Pathology and Oncology Research*.

[41] Lu Y, Xiong Y, Huo Y (2011). Grb-2-associated binder 1 (Gab1) regulates postnatal ischemic and VEGF-induced angiogenesis through the protein kinase A-endothelial NOS pathway. *Proceedings of the National Academy of Sciences of the United States of America*.

[42] Zhang QJ, Mcmillin SL, Tanner JM, Palionyte M, Abel ED, Symons JD (2009). Endothelial nitric oxide synthase phosphorylation in treadmill-running mice: role of vascular signalling kinases. *Journal of Physiology*.

[43] Jiang BH, Liu LZ (2008). AKT signaling in regulating angiogenesis. *Current Cancer Drug Targets*.

[44] Bian CX, Shi Z, Meng Q, Jiang Y, Liu LZ, Jiang BH (2010). P70S6K 1 regulation of angiogenesis through VEGF and HIF-1*α* expression. *Biochemical and Biophysical Research Communications*.

[45] Kong D, Yamori T (2008). Phosphatidylinositol 3-kinase inhibitors: promising drug candidates for cancer therapy. *Cancer Science*.

[46] Yu K, Lucas J, Zhu T (2005). PWT-458, a novel pegylated-17-hydroxywortmannin, inhibits phosphatidylinositol 3-kinase signaling and suppresses growth of solid tumors. *Cancer Biology and Therapy*.

[47] Kim DW, Huamani J, Fu A, Hallahan DE (2006). Molecular strategies targeting the host component of cancer to enhance tumor response to radiation therapy. *International Journal of Radiation Oncology Biology Physics*.

[48] Fei HR, Chen G, Wang JM, Wang FZ (2010). Perifosine induces cell cycle arrest and apoptosis in human hepatocellular carcinoma cell lines by blockade of Akt phosphorylation. *Cytotechnology*.

[49] Maffucci T, Piccolo E, Cumashi A (2005). Inhibition of the phosphatidylinositol 3-kinase/Akt pathway by inositol pentakisphosphate results in antiangiogenic and antitumor effects. *Cancer Research*.

[50] Romano S, Di Pace AL, Sorrentino A, Bisogni R, Sivero L, Romano MF (2010). FK506 binding proteins as targets in anticancer therapy. *Anti-Cancer Agents in Medicinal Chemistry*.

[51] Zhiyong C, Wentong L, Xiaoyang Y, Ling P PTEN's regulation of VEGF and VEGFR1 expression and its clinical significance in myeloid leukemia.

